# Physical symptoms and psychological distress among cancer patients: a moderated mediation model involving rumination

**DOI:** 10.3389/fpubh.2026.1853232

**Published:** 2026-06-30

**Authors:** Qiuxiang Sun, Weiping Chen, Yi Zuo, Junyi Wei, Mengjia Wang, Rui Zhang, Bin Wang, Qinghua Lu

**Affiliations:** 1 School of Nursing, Shandong Second Medical University, Weifang, China; 2 Oncology Department, Weifang Hospital of Traditional Chinese Medicine, Shandong Second Medical University, Weifang, China; 3 Integrated Neuropsychiatry Clinic, Shandong Mental Health Center, Shandong University, Jinan, China; 4 Department of Psychology, Shandong Provincial Hospital Affiliated to Shandong First Medical University, Jinan, China; 5 Department of Infection Management, Shandong Mental Health Center, Shandong University, Jinan, China

**Keywords:** cancer patients, moderated mediation model, physical symptoms, psychological distress, rumination

## Abstract

**Background:**

Cancer is a disease with a high fatality rate. In recent years, patients’ psychological problems associated with cancer have attracted much attention. Identifying the psychological distress of cancer patients, formulating and implementing psychological interventions, and satisfying the humanistic care needs of patients have become popular topics in cancer nursing research.

**Aims:**

The purpose was to investigate relationships among rumination, somatic symptoms and psychological distress in cancer patients and to explore the mediating role of rumination in the relationship between somatic symptoms and psychological distress.

**Methods:**

A total of 473 cancer patients admitted to the oncology department of a Grade A general hospital in Shandong Province from January 2023 to December 2023 were investigated via convenience sampling. The data were collected and examined via a general demographic data questionnaire, Ruminative Responses Scale (RRS), the Health Questionnaire-15 (PHQ-15) and the Kessler Psychological Distress Scale (K10).

**Results:**

The mean total psychological distress score in cancer patients was 21.77 ± 7.49. The 473 patients were divided into two groups for univariate analysis according to whether they experienced psychological distress (K10 ≥ 16). There were statistically significant differences in the marital status (*p* < 0.05), education level (*x*^2^ = 6.623, *p* < 0.05), cancer classification (*p* < 0.001), and symptom rumination (*x*^2^ = −13.737, *p* < 0.001) and brooding (*x*^2^ = −11.763, *p* < 0.001) subscores between the two groups. The reflective pondering subscore (*x*^2^ = −12.726, *p* < 0.001), total RRS score (*x*^2^ = −14.056, *p* < 0.001) and PHQ-15 score (*x*^2^ = −12.161, *p* < 0.001) were compared between the two groups. Psychological distress was positively correlated with the RRS score (*r* = 0.841, *p* < 0.05), the symptom rumination (*r* = 0.828, *p* < 0.05), brooding (*r* = 0.742, *p* < 0.05), and reflective pondering (*r* = 0.742, *p* < 0.05) subscores, and the PHQ-15 score (*r* = 0.743, *p* < 0.05). Rumination partially mediated the relationship between physical symptoms and psychological distress, with a mediating effect of 70.69%.

**Conclusion:**

There are close correlations among physical symptoms, rumination and psychological distress in cancer patients. Rumination plays a mediating role in the relationship between physical symptoms and psychological distress. Findings suggest that to alleviate patients’ psychological distress and improve their physical and mental health, medical personnel can not only address the physical discomfort symptoms of patients in time but also intervene in their negative cognitive thinking as observed by rumination.

## 1 Introduction

Cancer is a disease with a high mortality rate that has a great impact on patients’ physical and mental health and family happiness. According to the latest data from the Global Cancer Statistics 2020 (1), the global incidence and mortality of cancer are increasing rapidly. In 2019, there were 19.29 million new cancer cases worldwide and approximately 10 million deaths from cancer. Among them, 4.57 million new cancer cases have been reported in China, accounting for 23.7% of the world’s incidence, and 3 million deaths, which represents a 30% mortality rate. Cancer has seriously threatened the physical and mental health of people (2). The National Comprehensive Cancer Network (NCCN) defines psychological distress in cancer patients as a multifactorial, unpleasant emotional experience, including psychological, social, and physical experiences, which can affect patients’ ability to cope effectively with the disease (3). Studies have shown that cancer patients generally have a poor mental status, and the incidence of psychological distress ranges from approximately 24.2 to 76.0% (4). Approximately 27% of patients have moderate or greater psychological distress (such as anxiety, depression, fear, anger, loneliness, somatization disorders, etc.) after being diagnosed with cancer, and psychological distress may occur at different points across the disease course of cancer (5). Therefore, the mental health of cancer patients should be considered. At present, cancer patients’ psychological problems have attracted much attention. Identifying the psychological distress of cancer patients, formulating and implementing psychological care programs, and meeting the needs of patients for humanistic care are hot spots in cancer research (6).

Physical symptoms in cancer patients refer to the physical discomfort or dysfunction caused by the cancer itself or its treatment, which may vary depending on the type of cancer, stage, treatment regimen, and individual characteristics (7). Common physical symptoms in cancer patients include pain, cancer-related fatigue, digestive problems such as nausea, vomiting and loss of appetite, and sleep disorders (8). These physical symptoms not only directly cause discomfort to patients but also indirectly affect their mental health through cognitive and emotional mechanisms, resulting in emotional distress, such as depression and anxiety, reduced quality of life and social isolation due to fatigue, pain or treatment side effects (9). There is a two-way relationship between physical symptoms and psychological distress in cancer patients. Physical discomfort, such as pain and cancer-related fatigue, is related to the cancer itself or is a side effect of treatment and can also lead to emotional and psychological changes, such as anxiety, depression, and decreased self-esteem, in patients (10). The functional limitations of somatic symptoms can limit patients’ daily functioning, which in turn leads to feelings of helplessness and isolation, further exacerbating psychological distress (10). Studies have shown that anxiety and depression may reduce patients’ tolerance of pain and affect their ability to cope with the illness (11). Psychological distress can amplify a patient’s perception of physical symptoms, further aggravating physical symptoms. This interaction between physical symptoms and psychological distress creates a vicious cycle that significantly affects the overall recovery and quality of life of patients. Therefore, a framework for understanding the complex interplay between the physical and psychological symptoms of cancer patients has attracted attention. This is highly important for developing comprehensive intervention strategies and improving patient prognoses.

Studies have identified several factors associated with psychological distress, including resilience (12), rumination (13), pain (14), depressed mood (15), and sleep (16). According to reaction style theory (17), rumination is seen as a nonadaptive coping strategy for depressive emotional symptoms and their consequences. Rumination refers to the tendency of individuals who encounter negative life events to repeatedly analyze the situation, focusing on their own negative feelings and beliefs and failing to take positive actions to deal with these negative feelings (18). Treynor et al. (19) stated that rumination includes reflection and brooding. Reflection is an adaptive rumination component to deal with negative emotions and find solutions to problems, which is conducive to reducing depression. Rumination is often triggered by negative events, and individuals repeatedly think about their negative emotions and negative information, which causes anxiety, depression, and social disorders and prevents problem solving (20). Rumination can also cause inattention, hopelessness, suicidal ideation, poor sleep quality and weight loss (21).

The physical symptoms of cancer patients, such as pain, fatigue, nausea, vomiting and other uncomfortable physical feelings, directly cause patients to experience negative emotional reactions. Furthermore, patients will repeatedly think about the causes, consequences and treatment of their physical symptoms and cannot eliminate these negative thoughts (22). When patients become caught up in rumination, they may become overly focused on their body’s reactions, becoming overly alert and interpreting small physical changes as negative, thereby amplifying the perception of physical symptoms. Moreover, cancer patients may neglect to adopt positive coping strategies, such as seeking medical help and engaging in relaxation training, if they engage in compulsive rumination in response to physical symptoms (23). In addition, rumination may also lead to negative emotions such as anxiety and depression, which may themselves cause or aggravate certain physical symptoms, such as insomnia and loss of appetite (24). As a result, somatic symptoms may trigger rumination, causing the patient to think repeatedly about the symptoms and their negative effects, which in turn exacerbates psychological distress.

On the basis of the above research results, we found that in addition to the direct psychological distress caused by the physical symptoms of cancer, patients’ thought patterns about their symptoms also play important roles. There are other potential regulatory mechanisms at work in the relationship between physical symptoms and psychological distress. Therefore, we hypothesize that rumination modulates the relationship between physical symptoms and psychological distress. This study aims to explore the relationships among physical symptoms, rumination and psychological distress in cancer patients and further explore whether rumination plays a mediating role in the relationship between physical symptoms and psychological distress to provide a theoretical basis for intervention research to help cancer patients establish positive thinking patterns and alleviate psychological distress. Therefore, we propose the following hypotheses:

*H1*: The physical symptoms of cancer patients are positively correlated with psychological distress.

*H2*: Rumination in cancer patients is positively correlated with psychological distress.

*H3*: The physical symptoms of cancer patients are positively correlated with rumination.

*H4*: Rumination moderates the relationship between physical symptoms and psychological distress.

## 2 Materials and methods

### 2.1 Participants

From January 2023 to December 2023, convenience sampling was used to select cancer patients admitted to the Department of Oncology in a Class iii Grade A general hospital in Shandong Province for a questionnaire survey. The inclusion criteria were as follows: ① Hospitalized cancer patients with diagnostic evidence; ② full cognitive function and able to cooperate with the survey and communicate with the questionnaire personnel without obstacles; ③ aged 18–70 years; ④ signed the informed consent and agreed to participate in the survey voluntarily; ⑤ predicted survival time ≥3 months as judged by their doctors; and ⑥ complete disease history and treatment data. The exclusion criteria were as follows: ① patients with serious physical diseases; ② patients who did not consent to participate in the survey; ③ patients with cognitive and mental disorders; ④ illiterate patients.

In this study, according to Kendall’s sample size estimation principle (25), the sample size for cross-sectional studies is typically 10 to 20 times the number of variables. In this study, the demographic information questionnaire included 8 variables, and the three scales contained 5 variables. Considering a 20% rate of invalid questionnaires, the estimated sample size ranged from 156 to 312 participants and 531 cases were actually collected. After removing invalid questionnaires, the final sample size of the study was 473 cases. The effective recovery rate was 89.08%. All patients provided informed consent and voluntarily participated in this study, and the study was approved by the hospital ethics committee.

### 2.2 Questionnaire collection procedure

Upon receiving approval from the department head, the researcher proceeded to gather data within the department. Patients who fulfilled the specific inclusion and exclusion criteria were carefully selected and approached. The researcher then proceeded to provide detailed explanations to both the patients and their families regarding the purpose, methods, and significance of the study. Informed consent was obtained to ensure ethical practices were followed. To guarantee the reliability and accuracy of the questionnaire, the researchers thoroughly familiarized themselves with its contents prior to commencing the survey. They also provided active assistance to patients who faced challenges in reading or writing, ensuring that they were able to comprehensively understand and complete the questionnaire. Importantly, researchers refrained from engaging in any biased interference or influence.

The respondents were allocated a generous 20-min timeframe to complete the questionnaire. Upon completion, the questionnaires were collected anonymously. A meticulous examination was conducted to ensure that each questionnaire was fully completed without any missing sections. The collection process took place at the survey site.

### 2.3 Measures

#### 2.3.1 General demographic data questionnaire

The general information questionnaire was self-designed by the researchers and included the patient’s age, sex, marital status, place of residence, main caregiver, education level, monthly income and cancer type. According to previous studies on psychological distress among cancer patients, relevant demographic indicators were incorporated into this questionnaire (26).

#### 2.3.2 Kessler psychological distress scale (K10)

This scale was developed by Kessler et al. (27) and contains 10 items that evaluate the frequency of nonspecific psychological distress symptoms such as anxiety and depression experienced in the past 4 weeks. The Chinese version was tested for reliability and validity among university students by Zhou et al. (28). The Cronbach’s *α* was 0.8011, and the half-fold reliability was 0.7076. The scale uses a 5-point scoring system, with 1 point, 2 points, 3 points, 4 points and 5 points representing almost none, occasionally, some time, most of the time and all of the time, respectively. The total score is the sum of the scores of the 10 items and ranges from 10 to 50 points. The higher the total score is, the more severe the psychological distress. Individuals’ mental health status was divided into four levels on the basis of the total K10 score, with scores ranging from 10 to 15 indicating almost no psychological distress, scores ranging from 16 to 21 indicating mild psychological distress, scores ranging from 22 to 29 indicating moderate psychological distress, and scores ranging from 30 to 50 indicating severe psychological distress. A total K10 score higher than 16 points implied the presence of psychological distress. The Cronbach’s *α* of this scale in this study was 0.938.

#### 2.3.3 Ruminative responses scale (RRS)

The RRS scale was developed by Nolen-Hoeksema et al. (29). The scale assesses the frequency of rumination and behavior in the face of sadness or depression. It is a self-rating questionnaire consisting of 22 items, each of which is scored 1–4 points. The 22 items are divided into 3 dimensions: symptom rumination, brooding, and reflective pondering. Higher scores indicate more severe rumination. The scale has good test–retest reliability and internal consistency reliability. The Chinese researcher Liu et al. (30) carried out a relevant study on Chinese people using the RRS. The results showed that the quantitative reliability and validity of the scale were good. In this study, the Cronbach’s *α* of the symptom rumination, brooding and reflective pondering subscales were 0.904, 0.784, and 0.860, respectively, and the Cronbach’s *α* of the total scale was 0.949.

#### 2.3.4 Health Questionnaire-15 (PHQ-15)

This scale is a subscale of the Patient Health Questionnaire developed by Spitzer et al. (31) and is suitable for screening somatization disorders and assessing the severity of somatic symptoms. Kroenke et al. (32) reported that the PHQ-15 has good reliability and validity and it is widely used in medical institutions and scientific research. The scale uses a 0–2 three-level scale: 0–4: no physical problems; 5–9: mild physical problems; 10–14: moderate physical problems; and 15 points above: serious physical problems. A score above 10 suggests seeking help from a psychiatrist. The Cronbach’s *α* of the scale in this study was 0.887.

### 2.4 Statistical analysis

SPSS 25.0 was utilized for the statistical analyses. Measurement data with a normal distribution are represented as the mean and standard deviation. For the nonnormally distributed data, M (P25, P75) was used. Group comparisons were assessed using the x^2^ and *Z* tests. Spearman’s correlational analysis, hierarchical regression, structural equation modeling, R 4.1.2, and nonparametric percentile bootstrap test were used to analyze the relationships among physical symptoms, rumination, and psychological discomfort. The test level *α* = 0.05, *p* < 0.05 was considered statistically significant.

## 3 Results

### 3.1 General information

A total of 473 patients were included in this study; the age of the patients was 59.90 ± 8.03 years, 237 (50.11%) were younger than 60 years, and 236 (49.89%) were older than 60 years. There were 322 males (68.08%) and 151 females (31.92%); 464 (98.10%) were married, and 9 (1.90%) were unmarried, divorced, etc. There were 201 patients (42.49%) living in rural areas and 272 (57.51%) living in urban areas. There were 240 patients (50.74%) whose main caregiver was their spouse, 191 patients (40.38%) whose main caregiver was an immediate family member (parents, children), and 42 patients (8.88%) whose main caregiver was another patient. In terms of the patients’ educational background, 193 patients (40.80%) completed junior high school, 131 patients (27.70%) completed senior high school, and 149 patients (31.50%) completed college or above. The patients’ income level was as follows: ≤3,000 yuan for 106 patients (22.41%), 3,000–5,000 yuan for 238 patients (50.32%) and >5,000 yuan for 129 patients (27.27%). The cancer classifications were as follows: lung cancer in 147 patients (31.08%), liver cancer in 10 patients (2.11%), colon cancer in 88 patients (18.60%), esophageal cancer in 47 patients (9.94%), gastric cancer in 118 patients (24.95%), and rectal cancer in 63 patients (13.32%) (Table 1).

**Table 1:** Sociodemographic characteristics of the cancer patients.

Variables	*N*	Prevalence (%)
Age		
≤60	237	50.11
>60	236	49.89
Gender		
Male	322	68.08
Female	151	31.92
Marital status		
Married	464	98.10
Others (unmarried, divorced, etc.)	9	1.90
Place of residence		
Rural areas	201	42.49
Urban areas	272	57.51
Main caregiver		
Spouse	240	50.74
Immediate family members (parents, children)	191	40.38
Others	42	8.88
Educational background		
Junior high school	193	40.80
Senior high school	131	27.70
College or above	149	31.50
Monthly income (RMB)		
≤3,000	106	22.41
3,000–5,000	238	50.32
>5,000	129	27.27
Cancer classification		
Lung cancer	147	31.08
Liver cancer	10	2.11
Colon cancer	88	18.60
Esophageal cancer	47	9.94
Gastric cancer	118	24.95
Rectal cancer	63	13.32

### 3.2 K10, PHQ15 and RRS scores in cancer patients

The results of this study revealed that the mean total score of psychological distress in cancer patients was 21.77 ± 7.49. Among them, 161 (34.0%) had no psychological distress, 98 (20.7%) had mild psychological distress, 102 (21.6%) had moderate psychological distress, and 112 (23.7%) had severe psychological distress. The detection rate of psychological distress was 65.96% (Table 2).

**Table 2:** Composition of psychological distress severity of cancer patients.

Variables	*N*	Prevalence (%)
no psychological distress(10~15)	161	34.0
mild psychological distress(16~21)	98	20.7
moderate psychological distress(22~29)	102	21.6
severe psychological distress(30~50)	112	23.7
Total	473	100

The PHQ15 total score was 7.67 ± 0.43. The 15 items of the PHQ15 were ranked in order of the highest score, and the top five items were trouble sleeping (1.06 ± 0.05); feeling tired or having low energy (1.05 ± 0.05); nausea, gas, or indigestion (0.81 ± 0.06); constipation, loose bowels, or diarrhea (0.79 ± 0.06); and fainting spells (0.73 ± 0.05) (Table 3).

**Table 3:** Score of each item and total scores of PHQ-15.

Items	Mean ± SD	Rank
Stomach pain	0.41 ± 0.04	7
Back pain	0.35 ± 0.04	9
Pain in your arms, legs, or joints (knees, hips, etc.)	0.32 ± 0.05	10
Menstrual cramps or other problems with your periods (WOMEN ONLY)	0.16 ± 0.03	15
Headaches	0.24 ± 0.03	14
Chest pain	0.32 ± 0.043	11
Dizziness	0.37 ± 0.04	8
Fainting spells	0.73 ± 0.05	5
Feeling your heart pound or race	0.30 ± 0.04	13
Shortness of breath	0.46 ± 0.05	6
Pain or problems during sexual intercourse	0.31 ± 0.04	12
Constipation, loose bowels, or diarrhea	0.79 ± 0.06	4
Nausea, gas, or indigestion	0.81 ± 0.06	3
Feeling tired or having low energy	1.05 ± 0.05	2
Trouble sleeping	1.06 ± 0.05	1
Total scores of PHQ-15	7.67 ± 0.43	–

SD, standard deviation, PHQ-15 Rank 1–15 indicates the score of each item of PHQ-15 from highest to lowest.

The total RRS score was 40.77 ± 13.09, and the symptom rumination subscore was 21.95 ± 7.03, the brooding subscore was 10.10 ± 3.33, and the reflective pondering subscore was 8.73 ± 3.36 (Table 4).

**Table 4:** Score of each dimension and total scores of RRS.

Variable	Mean ± SD
Symptom rumination	21.95 ± 7.03
Brooding	10.10 ± 3.33
Reflective pondering	8.73 ± 3.36
Total RRS score	40.77 ± 13.09

SD, standard deviation; RRS, ruminative response scale.

### 3.3 Single-factor analysis of psychological distress in cancer patients

For the univariate analysis, 473 individuals were separated into two groups on the basis of whether they experienced psychological distress (K10 ≥ 16). The results revealed statistically significant differences in marital status (*p <* 0.05), education level (*x*^2^ = 6.623, *p* < 0.05*)*, cancer classification (*p* < 0.001), the symptom rumination(*Z* = −13.737, *p* < 0.001), brooding (*Z* = −11.763, *p* < 0.001), and reflective pondering (*Z* = −12.726, *p* < 0.001) subscores, and the RRS (*Z* = −14.056, *p* < 0.001)and PHQ-15 (indicating the physical problems of patients) (*Z* = −12.161, *p* < 0.001) between the two groups (Table 5).

**Table 5:** Single factor analysis of psychological distress in cancer patients (*n* = 473, Shandong, China).

Variables		K10<16 (*n* = 161)	K10 ≥ 16 (=312)	Test statistics	*p*
Age (in years)	≤60	71 (4.1%)	166 (53.2%)	3.522^a^	0.061
	>60	90 (55.9%)	146 (46.8%)		
Gender	Male	110 (68.3%)	212 (67.9%)	0.007^a^	0.934
	Female	51 (31.7%)	100 (32.1%)		
Marital status	Married	154 (95.7%)	310 (99.4%)	–	0.009
	Others	7 (4.3%)	2 (0.6%)		
Place of residence	Country	77 (47.8%)	124 (39.7%)	2.839^a^	0.092
	City	84 (52.2%)	188 (60.3%)		
Primary caregiver	Spouse	75 (46.6%)	165 (52.9%)	1.977^a^	0.372
	Parents and children	69 (42.9%)	122 (39.1%)		
	Others	17 (10.6%)	25 (8.0%)		
Educational background	Junior high school and below	76 (47.2%)^*^	117 (37.5%)^*^	6.623^a^	0.036
	High school/technical secondary school	46 (28.6%)	85 (27.2%)		
	College or above	39 (24.2%)^*^	110 (35.3%)^*^		
Monthly income	≤3,000 yuan	42 (26.1%)	64 (20.5%)	2.076^a^	0.354
	3,000–5,000 yuan	79 (49.1%)	159 (51.0%)		
	>5,000 yuan	40 (24.8%)	89 (28.5%)		
Cancer classification	Lung cancer	35 (21.7%)	112 (35.9%)	–	<0.001
	Liver cancer	0	10 (3.2%)		
	Colon cancer	44 (27.3%)	44 (14.1%)		
	Esophagus cancer	8 (5.0%)	39 (12.5%)		
	Gastric cancer	51 (31.7%)	67 (21.5%)		
	Rectal cancer	23 (14.3%)	40 (12.8%)		
RRS	Symptom rumination	16 (14, 18)	25 (19, 30)	−13.737^b^	<0.001
	Brooding	7 (6, 9)	11 (9, 13)	−11.763^b^	<0.001
	Reflective pondering	6 (5, 7)	10 (7, 12)	−12.726^b^	<0.001
	Total points	29 (27, 33)	46 (35, 54)	−14.056^b^	<0.001
PHQ-15		4(3, 5)	7 (5, 12)	−12.161^b^	<0.001

a : Chi-square test for categorical variables.

b : Mann–Whitney *U* test, with standardized *Z* values presented for non-normally distributed continuous variables.

“-” refers to the use of Fisher’s exact probability method without specific statistics; ^*^The pairwise comparison between sample rates was performed using the segmentation method; RRS, ruminative responses scale score; PHQ-15, patient health questionnaire-15; K10: psychological distress score.

### 3.4 Relationships among the PHQ15, RRS and K10 scores in cancer patients

**Figure 1:**
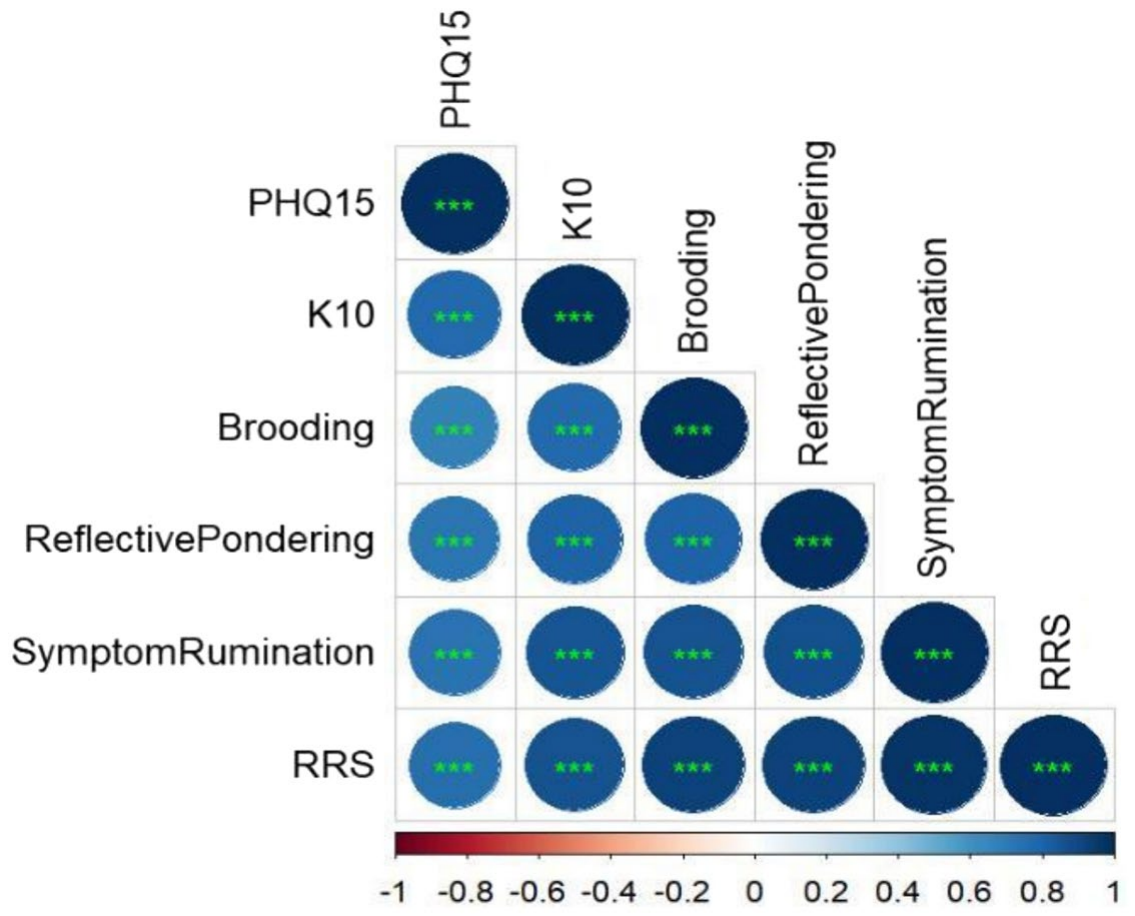
Correlation heat map of mental distress, somatic symptoms and rumination in cancer patients (*n* = 473, Shandong, China). RRS, ruminative responses scale score; PHQ-15, patient health questionnaire-15; K10: psychological distress score.

The RRS total score (*r* = 0.841, *p <* 0.05), symptom rumination (*r* = 0.828, *p <* 0.05), brooding (*r* = 0.742, *p <* 0.05), and reflective pondering (*r* = 0.742, *p <* 0.05) subscores, and PHQ-15 total score (*r* = 0.743, *p <* 0.05) were positively connected with psychological distress (Table 6). We constructed a correlation heatmap using R 4.1.2 to better depict the correlations among the indicators, as shown in Figure 1 (correlation heatmaps of the K10, PHQ15 and RRS scores).

**Table 6:** Correlation analysis of mental distress, physical symptoms and rumination in cancer patients (*n* = 473, Shandong, China).

Variables	Symptom rumination	Brooding	Reflective pondering	RRS	PHQ-15	K10
Symptom rumination	1					
Brooding	0.825**	1				
Reflective pondering	0.830**	0.774**	1			
RRS	0.969**	0.909**	0.901**	1		
PHQ-15	0.688**	0.594**	0.661**	0.694**	1	
K10	0.828**	0.742**	0.742**	0.841**	0.743**	1

**p < 0.05; RRS, ruminative responses scale score; PHQ-15, patient health questionnaire-15; K10: Psychological distress score; Rho, spearman rank correlation coefficient.

### 3.5 Analysis of the mediating role of rumination in the relationship between physical symptoms and psychological distress in cancer patients

It is widely believed that rumination plays a significant role in the relationship between physical symptoms and psychological distress. The potential mediating effect of rumination on this relationship has been a subject of investigation. To examine this mediating effect, an analysis was conducted on the basis of the study by Du et al. (33). Three separate regression analyses were carried out, with demographic factors serving as control variables, psychological distress serving as the dependent variable (Y), physical symptoms serving as the independent variable (X), and rumination serving as the mediator variable (M) (more information in Table 7).

**Table 7:** Hierarchical regression analysis of psychological distress in cancer patients (*n* = 473, Shandong, China).

Variables	K10	RRS	K10
Age (in years)	−0.815	−0.486	−0.616
Gender	−1.502*^b^*	−1.488	−0.894*^b^*
Marital status	−1.148	−3.547	0.302
Place of residence	−0.505	−1.175	−0.025
Primary caregiver	−0.545	−0.731	−0.246
Educational background	1.337*^b^*	2.720*^b^*	0.225
Monthly income	−1.401*^b^*	−2.081*^b^*	−0.550
Cancer classification	−1.408*^b^*	−0.434*^b^*	−0.231*^b^*
PHQ-15	1.271*^a^*	1.912*^a^*	0.489*^a^*
RRS	–	–	0.409*^a^*
*F*	82.958	72.880	165.543
*R^2^*	0.617	0.586	0.782
*△R^2^*	0.610	0.578	0.777
*p*	<0.001	<0.001	<0.001

^a^*p* < 0.01, ^b^*p* < 0.05; –: void item; RRS, ruminative responses scale score; PHQ-15, patient health questionnaire-15; K10: Psychological distress score.

**Figure 2:**
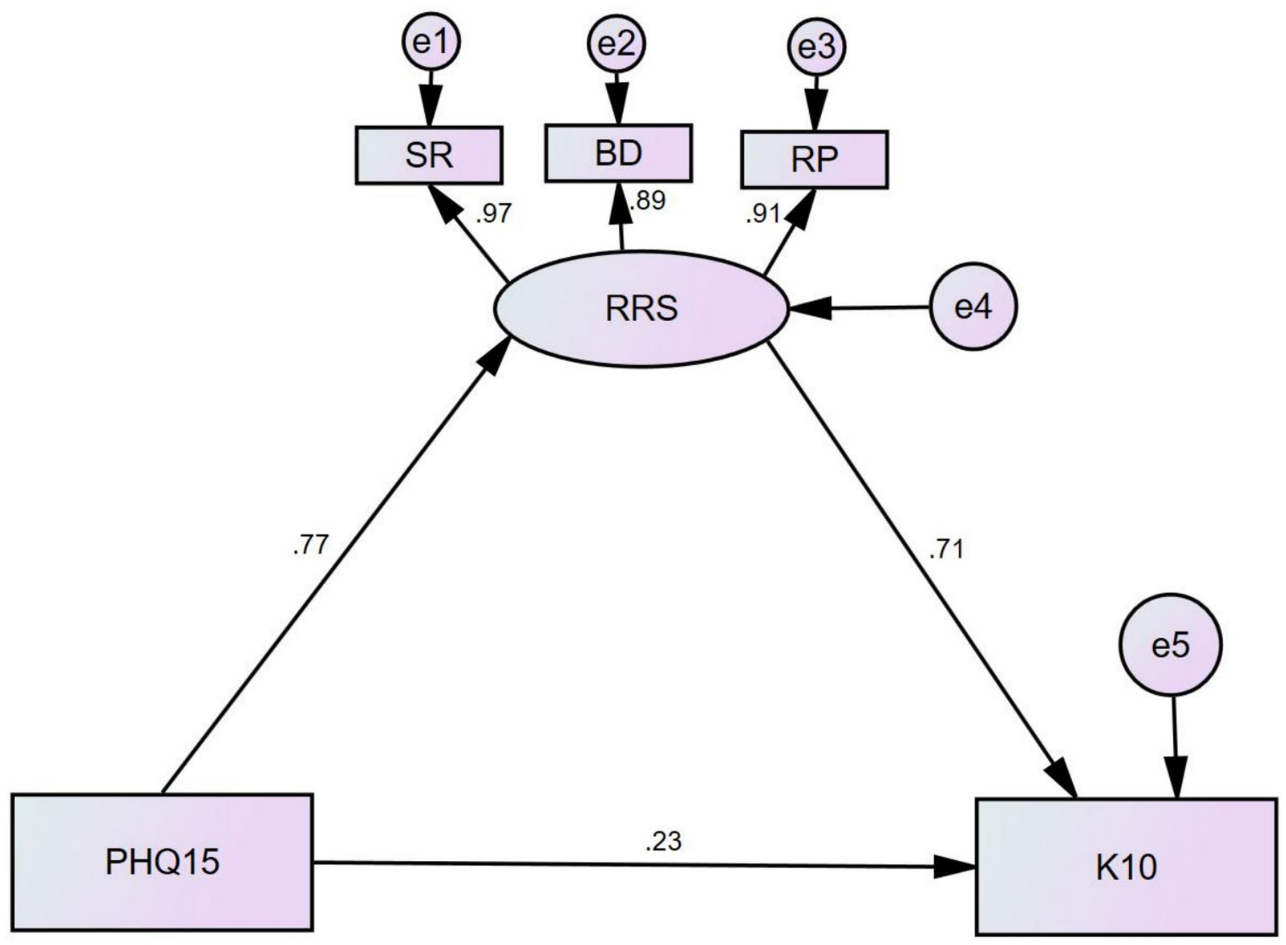
Mediating effect map of psychological distress in cancer patients (*n* = 473, Shandong, China). RRS, ruminative responses scale score; SR, symptom rumination; Bd, brooding; RP, reflective pondering; PHQ-15, patient health questionnaire-15; K10: Psychological distress score.

We built a model using structural equations to evaluate the associations among rumination, physical symptoms, and psychological distress in cancer patients on the basis of the findings of the stratified regression analysis. The model’s original fit factors were as follows: *X*^2^ = 9.461, root mean square error of approximation RMSEA = 0.054 (<0.08), normed fit index (NFI) = 0.996, comparative fit index (CFI) = 0.998, goodness-of-fit index (GFI) = 0.992, incremental fit index (IFI) = 0.998, adjusted goodness-of-fit index (AGFI) = 0.970 and chi-square/degrees of freedom (*X*^2^/DF) = 2.364 (<5). The relational model is shown in Figure 2 (mediating role of rumination in the association between physical symptoms and psychological distress). The data from the bootstrap test can be found in (Table 8), which includes information on the total effect, direct effect, and indirect effect of each pathway. The results indicates that rumination partially mediates the relationship, accounting for 70.69% of the total effect.

**Table 8:** Bootstrap test of psychological distress in cancer patients (*n* = 473, Shandong, China).

Variables	Effect	Bootstrap SE	*p*	Bootstrap 95% CI	
				LLCI	ULCL
Total effects	0.771	0.020	<0.001	0.728	0.807
Direct effects	0.225	0.037	<0.001	0.150	0.296
Indirect effects	0.545	0.033	<0.001	0.484	0.614

## 4 Discussion

### 4.1 Univariate analysis of psychological distress in cancer patients

This study demonstrated that married cancer patients have a higher incidence of psychological 269 distress, possibly because they have more family responsibilities, pressure to raise children, and 270 responsibility to support family members of older adults. Unmarried or divorced cancer patients have fewer family responsibilities and thus less psychological distress. This finding is consistent with the results of Cho et al. (34) study reporting that marital status is associated with cancer survival. However, the results of this study need to be further confirmed in large sample studies, since 98.1% of the participants in this study were married.

Furthermore, this study revealed that education level influences psychological discomfort in cancer patients. There was a statistically significant difference in the incidence of psychological distress between cancer patients with a high educational level (university or above) and a low educational level (below junior high school). This may be explained by the fact that persons with a greater level of education may have more complete consideration and a greater level of psychological demands, which leads to a greater level of psychological discomfort, which is also in accordance with the findings of Fountoulakis et al. (35).

The results of this study illustrate that cancer classification has an influence on psychological distress. The incidence of psychological distress in patients with lung cancer was the highest, which may be related to the fact that lung cancer has the highest incidence of malignant tumors and death. Wang et al. (36) reported that more than 80% of lung cancer clinical diagnoses are in the locally advanced stage and that more than 3/4 of diagnoses are in the late stage. In the middle and advanced stages of lung cancer, most patients no longer qualify for radical surgery, the disease progresses faster, the prognosis is poor, and the patients’ quality of life is low, which easily increases their psychological pressure and prompts psychological distress, such as anxiety and fear (37). This result is consistent with the results of Zabora et al. (38), who reported that the incidence of psychological distress in lung cancer patients is significantly greater than that in other types of cancer patients. These findings also suggest that healthcare providers should pay attention to the mental health of lung cancer patients. For patients diagnosed with lung cancer, especially those with advanced lung cancer, psychological distress and its severity should be evaluated in a timely and effective manner, and professional psychological intervention should be provided. Moreover, medical staff should strengthen the health education of patients and improve their understanding of lung cancer, chemotherapy and other related knowledge by using easy-to-understand language, aiming to overcome patients’ cognitive bias and alleviate psychological distress.

### 4.2 Research on the relationships among physical symptoms, rumination and psychological distress in cancer patients

This study revealed significant discrepancies in physical symptoms and rumination ratings between cancer patients with and without psychological distress, as well as pairwise positive associations among the three variables. According to the findings of this study, the median physical symptoms score in cancer patients was 6, and the majority of cancer patients had modest physical difficulties. Higher scores were associated with fatigue, sleep problems, constipation/diarrhea, nausea or indigestion, and weakness. Physical discomfort, such as pain and cancer-related fatigue, can be related to the cancer itself or a side effect of treatment. Moreover, owing to the continuous development of therapeutic drugs and diagnosis and treatment technology, the survival of cancer patients has significantly increased, but the duration of physical discomfort and the impact of psychological distress on patients has also increased (39). Wu et al. (40) reported that symptom discomfort in cancer patients is tightly associated with negative emotions such as worrying and sadness and simultaneously interacts with symptom distress and produces a vicious cycle. The impact of symptoms is common in cancer patients at different stages of the disease, especially during treatment. Moreover, as both the physical and psychological aspects of cancer are severely traumatizing, patients are prone to negative psychological symptoms, such as anxiety, depression, loneliness, and despair (41). According to Zhang et al. (42), the rate of depression in cancer patients is 76.5%, and the incidence of anxiety is 70.4%. Negative emotions such as anxiety and depression interact with symptom distress reciprocally, thereby changing patients’ bodily function and tolerance to treatment and seriously affecting quality of life (43). This finding also implies that clinical medical staff should offer cancer patients preventive education and guidance on diet and exercise, and they need to pay attention to adverse treatment reactions and address them in a timely and effective manner. Moreover, medical staff should pay attention to the occurrence of insomnia, fatigue and other symptoms in cancer patients; reduce the impact of symptom distress on cancer patients’ negative emotions; provide positive psychological interventions; improve rehabilitation self-confidence and self-esteem; reduce anxiety, depression and other negative emotions; and reduce the stress caused by physical and mental distress to improve patients’ quality of life.

The findings of this study’s univariate analysis revealed that cancer patients with or without psychological distress have statistically significant variations in the three characteristics of rumination. The results of the correlation analysis revealed that psychological distress was positively correlated with the three dimensions of rumination, possibly because rumination is a negative perception and processing method (17). This means that when acutely stressful life events occur, patients involuntarily, unconsciously and repeatedly pay attention to the negative changes in their behavior and thoughts and repeatedly think about the immediate negative emotional changes and the causes and results of such changes over a long period of time. This way of thinking can lead to maladaptive manifestations such as anxiety, despair, depression, and hesitation, which are not conducive to patients’ effective coping with the disease and treatment (44).

### 4.3 Analysis of the mediating effect of rumination on the relationship between physical symptoms and psychological distress in cancer patients

The results of the present study indicate that physical symptoms and rumination in cancer patients have positive effects on psychological distress. Research has shown that physical symptoms can not only directly affect the degree of psychological distress in patients but also indirectly affect psychological distress through rumination. Cancer patients may experience adverse reactions such as pain, fatigue, and nausea due to disease aggravation or adverse reactions to radiotherapy and chemotherapy during treatment. These physical symptoms may bother patients and affect their normal social functioning, thus influencing their quality of life. Both the aggravation and relief of symptoms change a patient’s psychological status and have an impact on their emotions, leading to anxiety and depression. In addition, the mediating effect of rumination between physical symptoms and psychological distress may result from negative self-referential processing (45), negative emotions and the persistence of rumination, which can impair an individual’s ability to solve problems and manage their emotions (17). Individuals in a state of rumination tend to associate negative information with themselves and overinterpret it (46). For example, when faced with negative life events such as cancer progression and adverse reactions to radiotherapy and chemotherapy, rumination will cause individuals to over reflect on their situation, deny their feelings and even potentially project their emotions into others’ views of themselves, causing them to believe that they are terrible people (47). Individuals with high rumination have difficulty extracting anything positive from the events they have experienced and believe that they are prone to fail the expected goals. This type of individual focuses on negative emotional experiences, which in turn aggravates their self-perception of uncomfortable physical symptoms, thereby further amplifying their cognitive bias of negative emotions and leading to aggravation of their psychological distress, including anxiety and depression (48). Therefore, in addition to timely treatment of cancer patients’ physical discomfort symptoms, clinical medical personnel can also help to change the thinking and cognitive mode of patients through interventions and reduce their excessive negative thinking, which will in turn reduce their psychological distress and promote their physical and mental health.

## 5 Implications for theory and practice

This study revealed that marital status, educational background, different cancer classifications, physical symptoms, rumination, etc., are influencing factors of psychological distress in cancer patients. These findings suggest that medical staff should pay more attention to patients with lung cancer, those with high education levels, those with physical symptoms, and those with higher RRS scores. The severity of physical symptoms was found to be closely related to psychological distress and vice versa. When cancer patients have sleep problems, gastrointestinal symptoms such as nausea and vomiting, fatigue and other symptoms of discomfort, they should be treated in a timely manner to reduce symptoms and relieve psychological distress. For cancer patients who ruminate, cognitive and behavioral psychological interventions should be carried out as soon as possible. Screening for psychological distress, such as anxiety and depression, is recommended routinely for cancer patients; a score that reaches a critical value (16 points) indicates that the risk of psychological distress in patients is high, and medical staff should increase their interventions. For such patients, professional consultation and treatment should be carried out in a timely manner; when the psychological distress score is close to the critical value, medical staff should pay attention to it and formulate a prospective intervention plan.

## 6 Conclusion

1. The detection rate of psychological distress in cancer patients was 66.0%. Marital status, education level, cancer classification, rumination, and physical symptoms differed among cancer patients with or without psychological distress.
2. Cancer patients’ physical symptoms, rumination and psychological distress were positively correlated, and there were mutually influential relationships among them.
3. Rumination played a mediating role in the relationship between physical symptoms and psychological distress in cancer patients.

## 7 Limitations

This study has several limitations. First, the cross-sectional design cannot establish causal links between variables, and single-site sampling also restricts the generalizability of conclusions. Second, all study indicators were obtained through self-reported scales, which may cause shared method variance and overestimated correlation results. Moreover, this research lacked a control group consisting of healthy individuals or patients with other chronic diseases, making it hard to verify the specificity of research findings. Additionally, critical clinical information including cancer stage, time since diagnosis and treatment status was not collected and analyzed in this study. In follow-up research, clinical evaluations and objective indicators will be adopted, control groups will be established for comparative analysis, and key clinical data such as cancer stage, disease duration and treatment phase will be comprehensively collected, so as to enhance the rigor and practical value of the research findings.

## Data Availability

The original contributions presented in the study are included in the article/Supplementary material, further inquiries can be directed to the corresponding author.
